# A clinician’s guide to understanding resistance to thyroid hormone due to receptor mutations in the TRα and TRβ isoforms

**DOI:** 10.1186/s40842-017-0046-z

**Published:** 2017-09-15

**Authors:** Brijesh K. Singh, Paul M. Yen

**Affiliations:** Laboratory of Hormonal Regulation, Cardiovascular and Metabolic Disorders Program, Duke-NUS Graduate Medical School, 8 College Road, Singapore, 169857 Singapore

**Keywords:** Resistance to thyroid hormone, Thyroid hormone receptors, Dominant negative activity, Thyroid stimulating hormone, Human mutation

## Abstract

There are two genes that express the major thyroid hormone receptor isoforms. Mutations in both these genes have given rise to Resistance to Thyroid Hormone (RTH) syndromes (RTHβ, RTHα) that can have variable phenotypes for mutations of the same receptor isoform as well as between the two receptor isoforms. In general, the relative tissue-specific distribution of TRβ and TRα determine RTH in different tissues for each form of RTH. These differences highlight some of the isoform-specific roles of each TR isoform. The diagnosis of RTH is challenging for the clinician but should be considered whenever a patient presents with unexplained elevated serum free T_4_ (fT_4_) and unsuppressed TSH levels, as well as decreased serum free T_4_/T_3_ ratio. Here we provide a guide for the clinician to diagnose and treat both types of RTH.

## Background

Fuller Albright first showed that pseudohypoparathyroidism represented a form of hormone resistance syndrome 75 years ago [1]. Since then, others have used clinical, biochemical, and molecular studies to identify many examples of hormone resistance with mutations in their corresponding receptors [18]. Indeed, hormone resistance due to mutations in many nuclear hormone receptors (NRs) such as the estrogen, glucocorticoid, peroxisome proliferator activator, and vitamin D receptors have been identified in affected individuals [53]. Similarly, numerous cases of resistance to thyroid hormone (RTH) and the corresponding mutations in the genes encoding human thyroid hormone receptors (TRs) have been reported.

In this current review, we will focus on a brief description of TRs and thyroid hormone (TH) action, as well as new clinical, biochemical, and molecular insights into RTH obtained from patients harboring mutations in the two TR isoforms, TRβ and TRα. After the recent identification of RTH in patients with mutations in the *THRA* gene, a new nomenclature was adopted to distinguish between types of RTH due to specific TR isoforms (please see below) [34]. The RTH syndromes due to TRβ and TRα are now called RTHβ and RTHα, respectively. Since RTHβ was identified and studied almost 50 years before the identification of RTHα (even though the precise mechanism for the former was not known at the time) [35], we will discuss RTHβ first. Mutations in the TH transporter, MCT8, and selenoprotein mutations that affect intracellular TH concentration but do not affect the function of TRs also have been identified. For more details on these syndromes, the reader is referred to several excellent recent reviews [15, 47]. New insights on the two forms of RTH have led to better understanding of the roles of the two TR isoforms on the function of different tissues as well as the regulation of the hypothalamic, pituitary, and thyroid (HPT) axis. Considering RTHβ and RTHα as potential diagnoses for abnormal thyroid function tests requires a rational approach for distinguishing these syndromes from other causes of inappropriate TSH secretion and low serum T_4_/T_3_ ratio, respectively, and will be discussed in more detail later in this article.

## Thyroid hormone action

THs are involved in the regulation of metabolism, proliferation, and growth of most tissues [5, 12, 28]. Serum TH levels are tightly controlled by the HPT axis to deliver appropriate amounts of TH to target tissues. The two major THs (T_3_ and T_4_) are iodothyrosines synthesized by the thyroid gland under the control of thyrotropin/thyroid stimulating hormone (TSH), a glycoprotein heterodimer that is produced by the pituitary gland. TSH, in turn, is regulated by thyrotropin releasing hormone (TRH), a tripeptide generated by the hypothalamus that is released into its own portal system to reach the pituitary. Both the production of TRH and TSH are under negative feedback control determined by the circulating free TH concentrations. Circulating THs, particularly T_4_, are mostly bound to transport proteins such as thyroxine-binding globulin (TBG), transthyretin (TTR), and albumin (HSA, human serum albumin). TBG binds 75% of serum T_4_ whereas TTR and HSA bind approximately 20% and 5%, respectively.

Although T_4_ is the major secreted form, T_3_ is significantly more potent than T_4_ and binds to TRs with 10-fold higher affinity [21, 25]. Thus, T_3_ is considered the active form of the hormone whereas T_4_ serves primarily as a less active precursor. After delivery to target tissues, THs utilize transporters (e.g., MCT8, MCT10, and OATP1C1) to cross the cell membrane and enter the cell [9]. THs then are metabolized by the iodothyronine deiodinases (Dio1, Dio2, and Dio3), a subfamily of selenoproteins [8]. The deiodinases serve as additional control points for TH action by regulating serum and intracellular TH concentrations. In particular, activation of TH is mediated by Dio1 and Dio2 conversion of T_4_ to T_3_ whereas inactivation of TH is regulated by Dio3 conversion to metabolites such as reverse triiodothyronine (rT_3_) and diiodothyronine (T_2_) [44].

## Thyroid hormone receptors

**Fig. 1:**
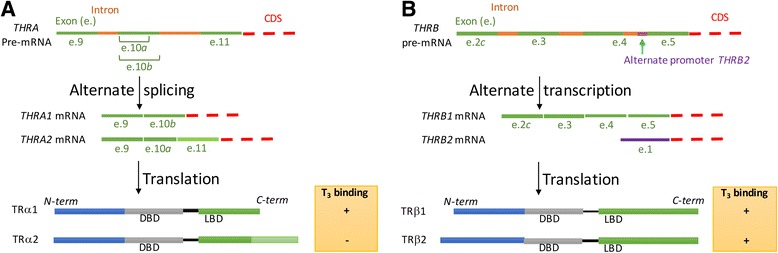
Alternative splicing and translation give rise to multiple TRα (**a**) and TRβ (**b**) isoforms

TRs belong to the nuclear receptor (NR) family that includes the steroid hormone, vitamin D, peroxisome proliferator activator, and retinoic acid receptors. Unlike peptide- or protein-binding receptors that are located on the cellular membrane, NRs are intracellular and bind to their cognate hormones either in the cytoplasm (steroid hormones) or nucleus (TH, vitamin D, retinoic acid) [53]. After binding to hormone, they have the ability to bind to hormone response elements (HREs) located in the promoter regions of target genes. As such, NRs can be considered hormone-inducible transcription factors. There are two major *THR* genes, *THRA* and *THRB*, that are expressed in a tissue-specific manner [12]. Two major *THR*A receptor splice variants (TRα1 and TRα2) are encoded by the *THRA* gene (Fig. 1a) and two major *THRB* isoforms (TRβ1 and TRβ2) are generated by alternate promoter choice on the *THRB* gene (Fig. 1b). TRα1 is highly expressed in the heart, bone, and skeletal muscle whereas TRα2 is widely expressed throughout the whole body. The alternative splicing of the *THRA* mRNA transcript leads to changes in the carboxy-terminus sequence of TRα2 that renders it incapable of binding to TH. It is possible that TRα2 may regulate alternative splicing of the *THRA* gene or may interfere with TRα1 action at the protein level. TRβ1 is predominately expressed in brain, liver and kidney whereas TRβ2 is found in the pituitary, retina, and cochlea. TRα1, TRβ1, and TRβ2 bind T_3_ with similar affinity.

**Fig. 2:**
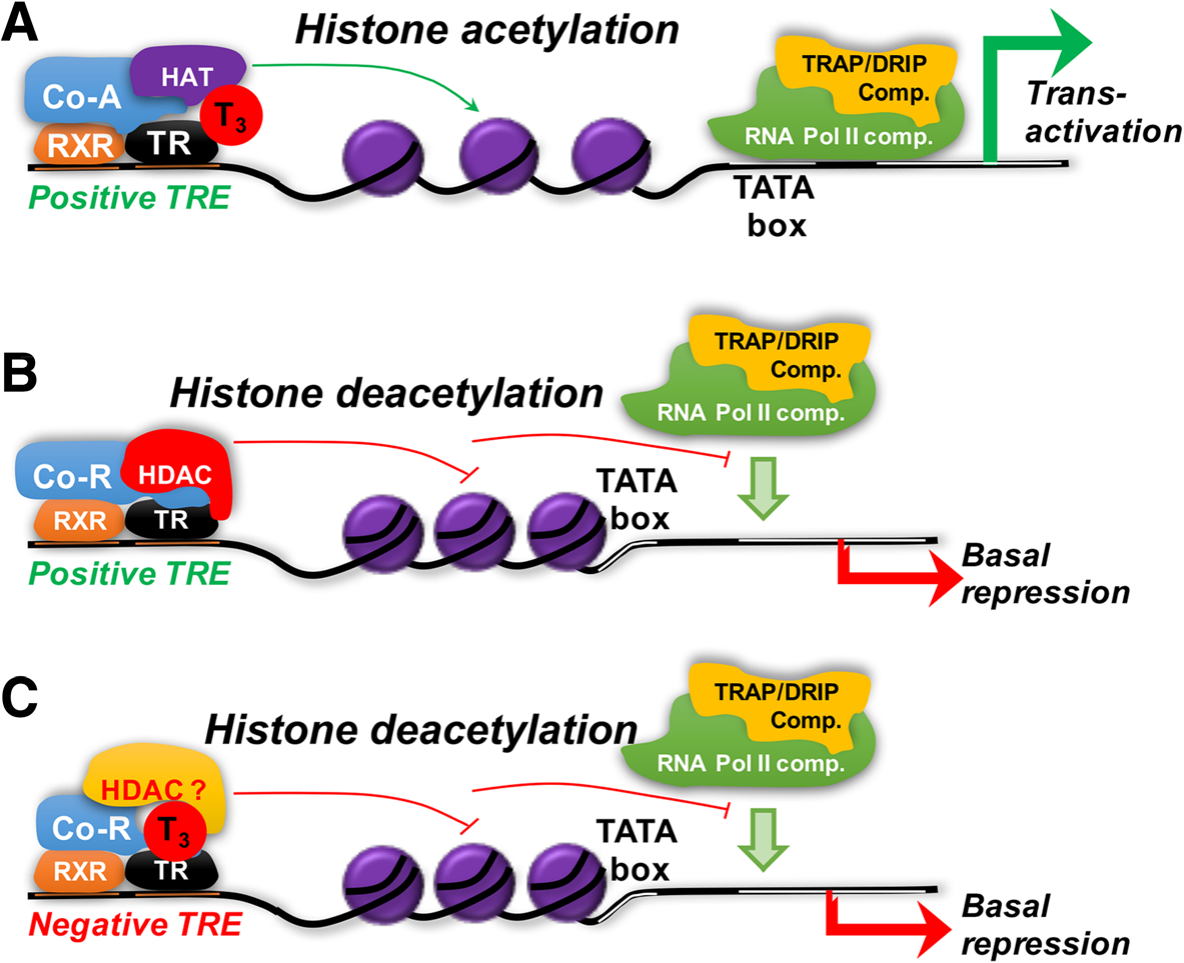
Role of co-activator and co-repressor recruitment in positively-regulated target genes. **a** For positively-regulated target genes, in the presence of T_3_, co-activators (Co-A) and histone acetyl transferases (HAT) are recruited by the T_3_-bound TR/RXR heterodimer sitting on the thyroid hormone response element (TRE). This leads to histone acetylation and chromatin nearby changes to a more open conformation to facilitate recruitment of RNA pol II to the TATA box region. Subsequently another co-activator complex, TH receptor-associated protein/vitamin D receptor interacting protein complex (TRAP/DRIP comp), is recruited by ligand-bound TR/RXR and RNA polymerase II complex to activate transcription. **b** For positively-regulated target genes in the absence of T_3_, TR/RXR has a different conformation than its T_3_-bound state, and has poor affinity for co-activator complexes. Instead, it recruits a co-repressor complex (Co-R) with histone deacetylase activity (HDAC). This leads to histone deacetylation and formation of a more closed chromatin conformation that does not allow RNA pol II binding to the promoter and thus “represses” transcription. **c** In some negatively-regulated target genes, in the presence of ligand, co-repressor and HDAC are recruited by TR/RXR sitting on the TRE. This leads to decreased histone acetylation and a more closed chromatin conformation that prevents RNA pol II binding to the promoter of the target gene, and thus negatively regulates transcription in the presence of T_3_. Please see text for more details

TRs have a modular structure, with a central DNA-binding domain and a C-terminal ligand-binding domain [5, 12, 28]. They typically will bind to DNA as heterodimers with another nuclear hormone receptor family member, retinoid X receptors (RXRs) (Fig. 2). These heterodimers can recognize specific DNA sequences, thyroid hormone response elements (TREs), located in the promoter regions of target genes. TREs typically are composed of two-half sites, most often organized as direct repeats, separated by 4 nucleotides (consensus DR4: 5′(A/G)GG(A/T)CANNNN(A/G)GG(A/T)CA 3′). TRs bind in a head to tail orientation with the upstream 5′ half site of DR4 bound by RXR and the downstream 3′ half site by TR. Interestingly, both unliganded and liganded TRs can bind to TREs; however, ligand binding to TRs induces conformational changes in the receptor that facilitate the recruitment of co-activators with histone acetyltransferase (HAT) and methyltransferase activity to induce conformational changes at specific chromatin sites in the promoters of positively-regulated target genes. These changes generate a permissive local chromatin environment that enables the binding and recruitment of the general transcriptional machinery (Fig. 2a) to the transcriptional start site and initiate transcription. In the absence of TH, TRs also can bind to TREs but they recruit co-repressors with histone deacetylase (HDAC) activity instead of co-activators/ HATs owing to their different conformation in the unliganded state. The co-repressor complex alters its surrounding chromatin structure by removing acetyl groups from histones to induce a conformational change in the histone structure that inhibits the binding of RNA polymerase II, and results in a decrease in target gene transcription (Fig. 2b). TREs can be located near or far from transcriptional start sites. The co-activator or co-repressor transcriptional complexes bound to them can interact co-operatively with multiple TR/TRE complexes in the promoter region to further regulate transcription. Taken together, this model suggests that TR/RXR heterodimer binding to the TRE and its recruitment of co-activators/corepressors play important roles in TH-mediated gene transcription (see below). Recently, using a method to examine TR binding throughout the whole genome, chromatin immunoprecipitation sequencing (ChIP-Seq), it was found that TRs can bind to DNA with sequences that do not resemble TREs and in non-promoter regions [4, 33]. Thus, it is likely that TRs interact with other transcription factors or chromatin via protein-protein interactions at these sites. There also is evidence that TH also may bind with low affinity to other non-TR proteins in the cell to mediate novel actions; but so far, these mechanisms are poorly understood [12].

The transcription of approximately half of all target genes is negatively regulated either indirectly (through the activation/increased expression of repressor transcription factors) or directly by TRs (Fig. 1c) [29]. Currently, the mechanism for negative regulation by TRs still is not well understood. *TSH*β and the *CGA* are two negatively-regulated target genes that are expressed in pituitary thyrotrophs. They generate two proteins, thyroid stimulating hormone β (TSHβ) and the common glycoprotein hormone α-subunit protein (α-GSU), that dimerize with each other to form TSH. Studies in pituitary-specific TR knockout mice suggest that the TRβ2 is the major isoform that controls the TH-mediated negative regulation of these target genes in the pituitary [51].

## Resistance to thyroid hormone β

### Clinical features

**Fig. 3:**
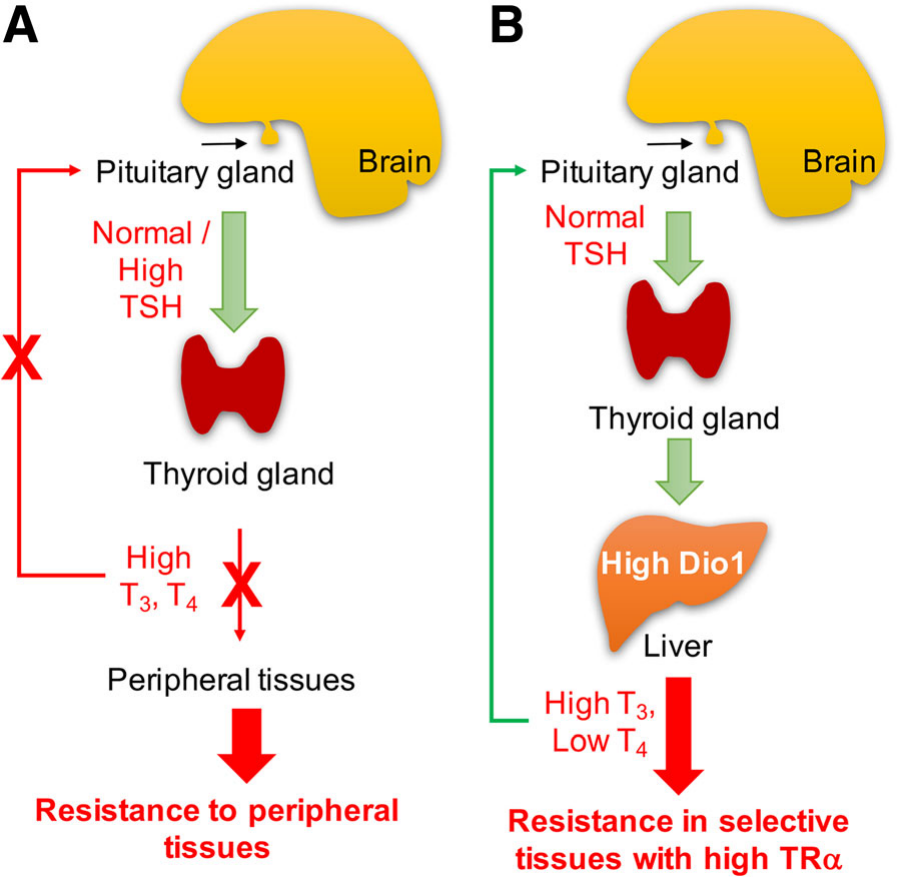
Models for RTHβ and RTHα. **a** RTHβ occurs in tissues expressing TRβ and causes a rise in serum T_3_ and T_4_ levels due to impaired negative feedback of the hypothalamic/pituitary/thyroid axis. **b** RTHα occurs in tissues expressing predominantly TRα (bone, gut, and heart) and causes symptoms shown in Table 1. In contrast to RTHβ, there is no defect in the negative feedback of the HPT axis by TH. However, patients have increased serum T_4_/ T_3_ ratios suggesting that a downstream effect such as increased deiodinase 1 (Dio 1) activity may occur

RTHβ is a rare disorder characterized by elevated levels of circulating free thyroid hormones, inappropriately normal or elevated TSH secretion, and decreased peripheral tissue responses to iodothyronine action (Fig. 3a) [7, 30, 36]. The incidence of RTHβ is estimated to be 1 case per 50,000 live births with affected individuals identified in Europe, Asia, and North and South America. So far, over 160 different mutations in TRβ have been found in RTHβ patients from more than 350 families. RTHβ follows an autosomal dominant inheritance pattern in families (80%) but also can be found sporadically in affected individuals with no other family history of RTHβ (20%) [36]. Patients with RTHβ typically have a heterozygous mutation in the *THRB* allele leading to the expression of a defective TRβ that has dominant negative activity on the transcriptional activities of the TRs encoded by the other normal *THRB* allele and the two normal *THRA* alleles [7, 36]. Major exceptions to this pattern were the first reported RTHβ kindred in which an autosomal recessive pattern of inheritance was observed [35]. The affected patients later were shown to harbor homozygous mutations in both *THRB* alleles that generated a severely truncated, non-functional form of TRβ [36].

In the clinical setting, RTHβ often is detected at childbirth during neonatal screening for congenital thyroid dysfunction when abnormal levels of T4, TSH, or both are identified, and further diagnostic testing undertaken. However, RTHβ also can go undetected due to its heterogenous presentation and variable symptoms [7, 36]. Goiter frequently is the main clinical finding that prompts the physician to order thyroid function tests and further investigations. In many cases, the high TH levels can compensate for tissue resistance; thus, affected individuals may appear to be clinically euthyroid. However, upon closer inspection, the compensation may be incomplete, and hypothyroidism is found in tissues that express predominantly TRβ (see below) such as the liver, kidney, and lung. Additionally, high endogenous TH levels sometimes can produce hyperthyroid effects, particularly in tissues that express predominantly TRα such as the heart and bone [7, 36]. These tissues do not express much mutant TRβ so it is likely that they are responding to the high circulating concentrations of TH [56].

In addition to goiter, the most common presenting signs and symptoms in patients with RTHβ are short stature, attention deficit disorder, and resting tachycardia although some patients may be entirely asymptomatic (Table 1). Moreover, the phenotypes and severity of TH dysfunction frequently vary among affected individuals expressing the same *THRB* mutation. Importantly, this variability in clinical phenotype may even occur among different affected family members with the same *THRB* mutation [7, 36]. These observations suggest that other genetic and epigenetic modifiers may affect the expression/penetrance of the RTHβ phenotype.

**Table 1:** Clinical features and diagnostic tests for RTHβ and RTHα

RTHβ	RTHα
Typical Clinical Features	
-Goiter	-Bradycardia
-Resting tachycardia	-Neurodevelopmental delay
-Osteoporosis	-Anaemia
-Short stature	-Skeletal dysplasia
-Attention deficit disorder	-Dysmorphia
-Family history (80%)	-Constipation
Diagnostic Tests	
-Increased fT_3_, fT_4_(Rule out antibody interference)	-Decreased T_4_/T_3_ ratio
-Normal/Elevated TSH	-Normal TSH
-Normal dialyzed free T_4_	-Exon sequencing of TRα
-Rule out autoimmune thyroiditis (anti-thyroid peroxidase, thyroglobulin, and TSH receptor antibodies)	
-Check serum markers TH hyperfunction (increased SHBG, ferritin, pro-collagen-1-N-terminal peptide (PINP) and decreased cholesterol in hyperthyroidism but normal in RTH)	
-Check serum a-GSU and compare with TSH (α-GSU (μg/l)/TSH (mU/l)] × 10 > 1.0 (suggests TSHoma)	
-Consider pituitary MRI (rule out TSHoma)	
-Exon sequencing of TRβ	

### Differential diagnosis

There are other clinical conditions of inappropriate TSH expression with increased serum T_4_, and they should be considered when attempting to make the diagnosis of RTHβ in a particular patient [36, 48]. First, there are several conditions or situations that can cause an *apparen* *t* increase in serum T_4_ with detectable TSH levels. These include: increased serum binding proteins (e.g.*,* thyroxine binding globulin), abnormal serum binding proteins with altered binding affinity for THs (e.g.*,* familial dysalbuminemic hyperthyroxinemia (FDH) and transthyretin variant), and anti-TSH or T_4_ antibodies. Measurement of serum free T_4_ levels, particularly by equilibrium dialysis and pre-clearance of anti-TSH/T_4_ autoantibodies before hormone measurements usually can distinguish these possiblities from RTHβ. Serum fT_3_ also should be normal in these cases. Additionally, it is important to evaluate family members for symptoms associated with RTHβ. Uncovering similar abnormalities in thyroid function tests among siblings and parents will provide important clues for the diagnosis of RTHβ since 80–90% case of RTHβ are familial.

Next, it is important to consider *transient* causes for elevated serum T_4_ and detectable TSH levels such as: systemic illness (sick euthyroid syndrome), acute psychiatric disorders, the neonatal period when there is a sudden burst of T_4_ release post-natally before full equilibration of the HPT axis, and early thyroxine replacement therapy in hypothyroid patients. Additionally, certain drugs can cause abnormal thyroid function tests that resemble those seen in RTHβ. Amiodarone, oral contrast agents, and β-blockers interfere with the conversion of T_4_ to T_3_ by inhibiting the enzymatic activity of Dio1. Serum TSH may be in the normal range in these patients. Amphetamines stimulate TRH release acutely leading to increased serum TSH and TH levels. Heparin induces lipoprotein lipase activity to increase serum free fatty levels that can interfere with TH binding to serum transport proteins.

The remaining other major cause for « inappropriate »TSH secretion with elevated serum T_4_ levels is TSH-secreting pituitary adenoma (TSHoma). Several important diagnostic tests are helpful for distinguishing between RTHβ and this condition: pituitary MRI (abnormal in TSHoma) and the common glycoprotein α-subunit hormone subunit (α-GSU) /TSH ratio. In the latter diagnostic measurement, there can be an inappropriately elevated secretion of common α-GSU in TSHomas such that the α-GSU /TSH ratio is elevated relative to TSH (α-GSU (μg/l)/TSH (mU/l)] × 10 > 1.0 in TSHomas) due to dysregulated over-secretion of α-GSU. However, this ratio may need to considered with caution when the circulating levels of other pituitary glycoproteins, particularly luteinizing hormone and follicle stimulating hormone, are elevated in post-menopausal women and can give a spuriously high ratio. Although not routinely used in the U.S. outside the academic setting, approximately 90% patients with RTHβ had normal or increased (similar to hypothyroid) TSH responses to TRH stimulation (200 μg bolus intravenously, sampling at 0, 20, 60, 90 and 120 min) whereas patients with TSHomas typically had high basal levels and only 39% responded to TRH [36, 41]. The reason for the occurrence of normal vs. increased TSH responses to TRH in patients with RTHβ may be that some patients have « compensated » pituitary response to the higher circulating TH levels whereas some patients do not, and thus have relative pituitary hypothyroidism. When TRH stimulation tests were performed after 3 days of T_3_ suppression at 50, 100, and 200 μg/days, euthyroid patients had suppressed TSH levels at 50 μg T_3_/day and almost all RTHβ patients also had some degree of TSH level suppression at 200 μg T_3_ /day, albeit to a lesser degree than euthyroid patients since most still had some residual TSH response at that dose of T_3_. In contrast, only 25% patients with TSHomas had any significant suppression of TSH levels after high dose T_3_ treatment [36, 41].

Measurement of metabolic markers of thyroid hormone action such as serum SGOT, SGPT, cholesterol, triglycerides, ferritin, osteocalcin, creatine phosphokinase (CPK), and sex hormone binding globulin (SHBG), also can be helpful in determining peripheral resistance. Serum prolactin can be elevated in patients with hypothyroidism and is increased in some patients with RTHβ, particularly those who previously were treated with ablative therapy. However, most RTHβ patients had normal basal prolactin levels (i.e.*,* without TRH stimulation). [36, 40] Among these markers, serum SHBG appears to be the one that is most reliably affected by decreased TH action. Serum SHBG levels in RTH patients are similar to those found in euthyroid patients but is significantly decreased when compared to thyrotoxic patients. Thus, a normal SHBG level in conjunction with elevated TH levels and unsuppressed TSH would be suggestive of RTH. Ferritin and osteocalcin levels are typically elevated in hyperthyroidism; thus, normal levels also would be supportive of RTH. SGOT, SGPT, cholesterol, and triglyceride levels are responsive to TH but are nonspecific for hyperthyroidism, and thus may have limited utility. Additionally, TH effects on the neuromuscular system also can be assessed by measuring serum CPK concentration (elevated in RTH), and performing careful neurological examination looking for signs of hypothyroidism. Finally, if clinical and laboratory evidence support the diagnosis of RTHβ, a direct sequencing of the *THRB* gene exons, particularly those sequences that encode the LBD should be considered (see below). Identification of the mutation may be useful for future prenatal diagnosis of RTHβ. Specific TRβ mutation testing is available commercially from Quest Diagnostics (Madison, NJ) and several companies offer whole exome sequencing (e.g.*,* Macrogen (Rockville, MD), Otogenetics (Atlanta, GA), and GATC (Constance, Germany)). Finally, a letter with a clear explanation of the diagnosis should be provided to the patient and be presented to any physican taking care of the patient in order to prevent inappropriate treatment for elevated serum T_3_ or T_4_.

### TRβ Mutations and mechanism

In both familial and sporadic cases of RTHβ, TRβ point mutations cluster in the 3 major “hot spots” of the LBD [13, 17, 38]. In familial RTHβ, affected members have one normal and one abnormal *THRB* allele, consistent with the autosomal dominant pattern of inheritance seen in these families. In sporadic RTHβ mutations, similar findings in the *THRB* alleles also are observed. Since TRβ mutations occur in the LBD, they often lead to decreased T_3_-binding affinity. So far, no germ line mutations have been identified in the DBD or N-terminal regions of TRβ. In patients with RTHβ, most mutations are nucleotide substitutions that result in single amino acid changes. However, nucleotide deletions or insertions that cause single amino acid deletions and frameshift mutations, and premature stop codons also have been reported. Interestingly, the first described case of RTHβ occurred was inherited in a recessive pattern, and later shown to be due to complete from the exon-coding region resulting in absence of TRβ [42]. Since DNA-binding is required for the autosomal dominant inheritance in RTH, it is possible that mutations in the amino-terminus or DNA-binding may have a recessive phenotype. The inability to find mutations in these regions, suggests that if they exist, they may have little or no distinctive phenotype suggesting TH dysfunction. It is noteworthy that so far, no LBD mutations have been found that increase T_3_-binding affinity or its transcriptional activity.

**Fig. 4:**
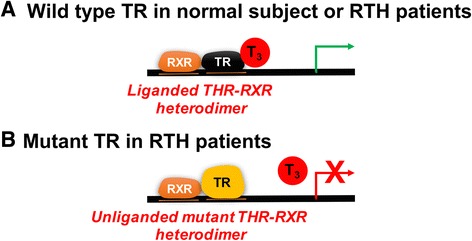
Model for resistance to thyroid hormone in RTHβ patients. **a** In both normal and RTH patients, wild-type TRβ and TRα isoforms derived from normal *THRB* and *THRA* alleles bind as TR/RXR heterodimers to the TRE and are able to activate transcription. **b** The mutant TRβ encoded by the abnormal *THRB* allele in RTH patients bind to the TRE constitutively in both the presence and absence of T_3_. Since it has decreased ligand-binding affinity, its ability to recruit co-activators and activate transcription is impaired. The unliganded mutant TR/RXR heterodimer thus competes with T_3_-bound wild type TR/RXR heterodimer for binding to the TRE

At the molecular level, mutant TRβs have decreased transcriptional activity due to their reduced ligand-binding affinity. They also can competitively block normal TRs from binding to TREs since they generally retain their DNA-binding capability [54]. This interference of normal TR function by the mutant TRβ (so called “dominant negative effect”) leads to decreased overall transcriptional activity in target genes (Fig. 4) [30, 52]. Further support for this model comes from studies showing loss of dominant negative activity by mutant TRβs in which a second mutation was introduced into the DBD to abrogate DNA binding [27]. Moreover, it is likely that unliganded TR/co-repressor complex needs to leave the TREs in the presence of TH before liganded TR/co-activator complex can bind to the TREs and activate transcription. In this connection, constitutive binding of mutant TRs to TREs, combined with decreased corepressor dissociation and coactivator recruitment prevent normal T_3_-bound TRs from binding to TREs and activate transcription of target genes [26, 39]. Together, these effects likely are the main contributors for the dominant negative inhibition on transcription of target genes by mutant TRs. In general, the severity of T_3_ binding impairment by mutant TRs correlates with the severity of clinical phenotype, although there are some exceptions [7].

### Treatment

In most patients, RTHβ appears to be adequately compensated by the increased endogenous supply of TH reflected by the increased serum fT_3_ and fT_4_ levels and normal or near normal TSH levels. These patients appear clinically euthyroid and eumetabolic [50]. Special care must be made not to misdiagnose and inappropriately treat these RTHβ patients for “hyperthyroidism” because of the high serum TH levels [49]. Unfortunately, some patients with RTHβ have undergone unnecessary radioactive iodine ablation, thyroid surgery, or anti-thyroidal medical treatment (e.g.*,* propylthiouracil, carbimazole) based upon the presumption of hyperthyroidism. These inappropriate treatments led to worsened symptoms, as patients were rendered more hypothyroid in resistant tissues despite normalization of serum TSH and T_4_ levels. Likewise, patients with compensated RTHβ do not require additional thyroxine treatment despite their RTHβ. Such treatment should only be considered in uncompensated RTHβ patients that have undergone thyroid ablation or surgery and have limited or no thyroid reserve or have decreased thyroid function due to autoimmune disease. Previous thyroid function test results when the patient was in the “compensated” baseline state before surgery or thyroid injury can be extremely useful for determining the optimal replacement dose in these RTHβ patients level, and can be used to follow patients’ responses to treatment.

The possibility of uncompensated RTHβ in de novo cases should be suspected if patients have TSH levels higher than normal levels, together with elevated serum fT_3_ and fT_4_ levels. Thus, elevated TSH levels without any signs or symptoms of hypothyroidism should raise the suspicion of possible RTHβ, and serum fT_3_ and fT_4_ levels obtained if they were not measured during an initial screening. In patients with previous thyroid surgery or Hashimoto’s thyroiditis, uncompensated RTHβ might be suspected if unusually high replacement doses of levothyroxine are necessary to reduce the elevated TSH levels. Finally, in some cases of uncompensated RTHβ, patients may be asymptomatic or have complaints suggestive of hypothyroidism while exhibiting paradoxically high serum fT_3_ and fT_4_ levels and a TSH level that is above the normal range.

The assessment of uncompensated RTHβ also is made clinically, in conjunction with laboratory tests that suggest peripheral resistance (e.g.*,* decreased serum SHBG). However, RTHβ may manifest itself in children by decreased and/or delayed growth or failure to gain weight. When RTHβ is not compensated, thyroxine can be given in incremental doses with simultaneous monitoring of parameters linked to TH action such as liver function tests, CPK, SHBG, PRL, and TSH until normal levels are achieved. In children, levothyroxine has been used under close supervision to improve growth and school performance; however, the results, have been variable. The presence of tachycardia should not be a reason to withhold treatment for uncompensated RTHβ as it can be managed by concomitant administration of a β-adrenergic blocker such as atenolol.

Recently, TRβ-specific analogs that have higher affinity for TRβ than TRα have been developed [6]. These drugs primarily are aimed as potential therapies for hypercholesterolemia, obesity, and diabetes. However, it is possible that these drugs may also be useful in patients with RTHβ. In this connection triiodothyroacetic acid (TRIAC) has been used to treat patients with RTHβ; however, there have been no studies thus far comparing the effectiveness of TRIAC vs. levothyroxine for the treatment of uncompensated RTHβ [32].

## Resistance to thyroid hormone α (RTHα)

### Clinical features

Previous studies in genetic models of RTHα such as TRα knockout mice that do not express TRα and mutant TRα knock-in mice that express a TRα mutation in the *THRA* gene locus, suggested that lack of TRα or expression of an inactive TRα were not lethal [16]. Surprisingly, these genetic perturbations caused only relatively mild hypothyroid-like symptoms, particularly in the heart and bone. The prevalence of RTHα in man is not known but it is possible that this disorder has not been adequately recognized clinically since it lacks a distinctive phenotype and also may be associated with unusual phenotypes such as autism spectrum disorder. An examination of large databases showed approximately 100 non-synonymous variants in *THRA* in 60,000 exomes; however, only a small number of these variants were mutated at homologous TRα sites and would be expected to give a distinct phenotype [24].

RTHα in man was first described in a 6-year-old girl with skeletal dysplasia, bradycardia, growth retardation, neurodevelopmental delay, and constipation. Interestingly, the patient harbored a TRα mutation (Glu403X) that led to a frameshift mutation as well as loss of helix 12 in the LBD due to the introduction of a premature stop codon. This mutation decreased both its ligand binding affinity for TH and its transcriptional activity similar to the TRβ mutations found in RTHβ [10, 24]. This individual had borderline low or normal T_4_, borderline high or normal T_3_, and normal TSH concentrations in her serum. Shortly afterwards, several adult male and female individuals were identified that harbored frameshift/premature stop mutations within the TRα1 LBD [14, 45]. These patients also had additional features in their phenotypes such as macrocephaly, anemia, and dysmorphic facies. Based upon the reports of nearly 30 patients with RTHα [14, 23, 24, 43], clinical features that are most commonly found among RTHα patients include bradycardia, constipation, reduced and delayed bone growth, delayed psychomotor development, decreased metabolic rate, as well as skeletal abnormalities manifested by delayed fusion of epiphyses and reduced bone growth (Table 1). Additionally, dysmorphic features have been reported in some affected individuals from several kindreds, and they include: macrocephaly, late fontanelle closure, dysmorphic and broad facies, flattened nose, enlarged tongue, and thickened lips. Of note, many of these features can resemble those found in congenital and primary hypothyroidism. Additionally, the tissues associated with these features contain mostly TRα, and thus would be expected to be “hypothyroid” with respect to TH action due to the dominant negative effect by mutant TRα. Interestingly, several cases of RTHα also were identified after screening for abnormal thyroid function in patients with dysmorphic features [24]. On the other hand, there can be large variation in the severity of the phenotypes in RTHα as some patients can have mild phenotypes with minimal symptoms [14]. When RTHα patients are compared with RTHβ, it appears that they can present with a wider repertoire of phenotypes than RTHβ as well as exhibit phenotypes that are distinct from RTHβ.

RTHα patients typically have increased/high-normal T_3_ and decreased/low-normal serum T_4_ levels, resulting in a markedly reduced T_4_/T_3_ ratio (Fig. 4a). Low serum rT_3_ levels also have been reported in some cases. Of note, serum TSH levels are usually normal. The reason for the low T_4_/T_3_ ratio in affected individuals is not known; however, it is noteworthy that increased hepatic DIO1 expression was observed in a dominant negative TRα knockin mouse model [55] so it is possible that increased conversion of T_4_ to T_3_ may be involved in generating this serum TH profile. Additionally, TRα is highly expressed in the skin so RTHα in that tissue could lead to decreased DIO3 expression and activity, and thus lead to accumulation of serum T_3_ [11].

### Differential diagnosis

Although rare, RTHα should be considered in the differential for children with decreasted growth rate, dysmorphic features, and delayed psychomotor development. It also should be considered in adults with a similar previous history as well as in patients with unexplained constipation, megacolon, and bradycardia [24, 46]. The low serum T_4_/T_3_ level is a distinctive and consistent feature in RTHα that can help identify potential cases. Of note, this biochemical abnormality also can be seen in disorders involving decreased TH synthesis since T_4_ is the major form of TH that is synthesized and released by the thyroid gland. Thus, congenital hypothyroidism or environmental causes of hypothyroidism (e.g.*,* iodine deficiency) can exhibit this serum TH profile. Additionally, patients with Allan–Herndon–Dudley syndrome, a condition in which patients harbor a mutation in one of the major TH transporters, MCT8, can present with a similar TH profile [15]. However, these patients have severe mental retardation and progressive spastic paralysis as well as an x-linked inheritance pattern so it is relatively easy to distinguish them from patients with RTHα based upon their clinical features.

### Mechanism

In patients with RTHα, TRα mutations in the LBD due to nucleotide substitutions that cause missense amino acid changes, deletions, or insertions, as well as frameshift/premature stop mutations have been described [14, 24, 43]. Of note, none of the *THRA* mutations described so far involve the exon regions or the expression of the *REV-ERB*α, a gene that is transcribed from the opposite strand of the *THRA* locus. Heterozygous *THRA* mutations are found in both sporadic and familial RTHα. Thus, the molecular mechanism for RTHα is similar to RTHα, by virtue of the expression of the mutant TRα from one *THRA* allele and a normal TRα from the other *THRA* allele, and normal TRβs from the two *THRB* alleles [24]. The mutant TRα has “dominant negative activity” on normal TRs expressed within the cell. The degree of dominant activity depends upon the relative amount of mutant TRα expressed within a particular cell as well as the residual ligand-binding and DNA-binding affinities of the mutant TRα.

Mutant TRαs bind to T_3_ with decreased affinity or fail to bind ligand; and thus lead to decreased or no transcriptional activity, respectively. Similar to TRβ mutations in RTHβ, TRα1 mutants inhibit the function of normal TRs in a dominant negative manner when they are co-expressed in transfected cells. In support of this mechanism in affected individuals, expression of TH-responsive target genes are blunted in peripheral blood mononuclear cells of a patient with RTHα [24], suggesting that mutant TRαs can exert dominant negative activity in vivo (Fig. 4). Additionally, studies have shown that many of the naturally occurring TRα mutations have decreased release of NCoR due to lower T_3_ binding affinity by mutant TRαs.

### Treatment

In adults with RTHα, titrating the appropriate levothyroxine dose is difficult. Heart rate and cardiac contractility can remain blunted despite thyroxine therapy. Excessive thyroxine treatment to correct cardiac parameters also may lead to undesirable toxicities in tissues that express predominantly TRβ such as the liver. Interestingly, thyroxine therapy does not ameliorate the anaemia observed in RTHα patients. In children, the treatment of RTHβ is challenging [24, 46]. TH induces the expression of insulin-like growth factor 1 (IGF1) and sex hormone binding globulin (SHBG) and decreases the production of cholesterol and triglycerides. Thus, thyroxine therapy can improve overall height and bone growth in RTHα [23, 24]. Of note, growth hormone in combination with thyroxine to increase IGF1 has not led to significant improvement in height and growth [24]. Thyroxine therapy also can improve the constipation symptoms commonly found in children with RTHα.

Thyroxine therapy suppresses serum TSH levels and increases fT_3_ above normal levels. Serum SHBG, which is induced by TH in the liver, as well as bone turnover markers also can increase above normal levels, most likely due to increased TH activity in tissues and cell types that express mostly TRβ Just as in the case for RTHβ, development of TRα1-selective thyromimetics may be helpful to selectively activate normal TRα1 and/or mutant TRα1 with weak binding affinity for T_3_ to overcome TH resistance in tissues that express predominantly TRα. Another potential therapeutic strategy is to develop drugs that enable nuclear receptor co-repressor (NCoR) to dissociate from unliganded TR or to abrogate the activity of histone deacetylases recruited by NCoR. In this connection, an inhibitor of histone deacetylase, suberoylanilide hydroxamic acid improved some of the phenotypic abnormalities of RTHα such as delayed and decreased growth and bone development in a mouse model of RTHα [20].

## RTH in patients without TRβ mutations

Several patients with RTH have been identified who do not have TRβ or TRα mutations [31, 37]. Additionally, no mutations in various candidate co-factors involved in TR-mediated transcription were found. It is likely that epigenetic effects that alter the expression of various genes involved in transcription may be involved, although it has not been investigated in these patients so far.

## Somatic TR mutations

Somatic TRα and TRβ mutations have been identified in human hepatic, thyroid, and renal cell cancers [19, 22] in addition to TSH-secreting pituitary adenomas [2, 3]. These findings suggest that TR mutations likely contribute to RTH in these tumors; however, they are not sufficient to cause oncogenesis since RTH patients with germline TRβ mutations do not appear to have an increased risk for cancer.

## Conclusion

Although RTHβ and RTHα are rare genetic disorders that cause RTH, they need to be considered when patients present with enigmatic thyroid function tests. In particular, when patients present with high free T_3_ and T_4_ with non-suppressed TSH levels (RTHβ) or reduced free T_4_/ free T_3_ ratio with normal TSH level in the serum (RTHα). Associated with each condition are some characteristic features in their phenotype that also highlight the isoform-specific expression and particular roles of TRβ and TRα. The clinical spectrum for both RTHβ and RTHα is wide and heterogenous; moreover, there can be variable phenotypes in patients with the same mutations. These observations suggest that genetic and epigenetic modifiers likely play important roles in the phenotypes of affected individuals. The identification of TR mutations as causes for the two forms RTH, elucidation of their mechanism for causing resistance, correlation of genotype with phenotype, and the development of criteria for clinical diagnosis and treatment of RTH provide elegant examples of the convergence of basic, translational, and clinical research to improve the understanding and management of a genetic endocrine disorder.

## Abbbreviations

ChIP-Seq: chromatin immunoprecipitation sequencing
FDH: familial dysalbuminemic hyperthyroxinemia
fT_3_: serum free T_3_ concentration
fT_4_: serum free T_4_ concentration
HAS: human serum albumin
HAT: histone acetyltransferase HREs hormone response elements
HPT: hypothalamic, pituitary, and thyroid
IGF1: insulin-like growth factor 1
NCoR: nuclear receptor co-repressor
NR: nuclear receptor
rT_3_: serum reverse triiodothyronine concentration
RTH: resistance to thyroid hormone
RTHα: resistance to thyroid hormone receptor α
RTHβ: resistance to thyroid hormone β
RXRs: retinoid X receptors
SHBG: sex hormone binding globulin
TBG: thyroxine-binding globulin
TH: thyroid hormone
*THRA*: thyroid hormone receptor β gene
*THRB*: thyroid hormone receptor β gene
TR: thyroid hormone receptor
TREs: thyroid hormone response elements
TRH: thyrotropin releasing hormone
TRIAC: triiodothyroacetic acid
TRα: thyroid hormone receptor α
TRβ: thyroid hormone receptor β
TSH: thyrotropin/thyroid stimulating hormone
TSHoma: TSH-secreting pituitary adenoma
TSHβ: thyroid stimulating hormone β subunit
TTR: transthyretin
α-GSU: common glycoprotein hormone α-subunit protein
